# Prevalence of smell and taste dysfunction in a cohort of CoVID19 outpatients managed through remote consultation from a large urban teaching hospital in Dublin, Ireland

**DOI:** 10.1016/j.infpip.2020.100076

**Published:** 2020-07-15

**Authors:** Colm Kerr, Gerry Hughes, Louise McKenna, Colm Bergin

**Affiliations:** a Department of Infectious Diseases, St. James's Hospital Dublin, Ireland; b School of Medicine, University of Dublin Trinity College, Dublin, Ireland

**Keywords:** SARS-CoV-2, CoVID19, Smell, Taste, Anosmia

## Abstract

**Background:**

Coronaviruses are known to precipitate disorders of smell and taste function. With the emerging global coronavirus disease (CoVID19) pandemic due to the severe acute respiratory syndrome coronavirus 2, early reports suggested that smell and taste dysfunction were clinical features of CoVID19. Our study aimed to investigate the prevalence of smell and taste disturbances in a cohort of CoVID19 positive patients who were isolated at home and being medically managed through telephone consultation.

**Methods:**

This was a retrospective cross-sectional study conducted at St. James’s Hospital Dublin, an urban 850 bed tertiary referral centre. 46 out of 50 CoVID19 positive patients, managed through a telephone clinic from the hospital infectious disease department, were assessed for olfactory and gustatory function loss.

**Results:**

The median age of participants was 36.5 years (interquartile range (IQR) 27 - 48) and 19 (41%) were male. The majority (31; 67%) never smoked and 17 (37%) reported co-morbidities. Approximately half of patients reported some degree of smell (22; 48%) or taste (25; 54%) function loss. 13 patients (28%) reported a complete loss of sense of smell, 8 (17%) reported a complete loss of sense of taste and 7 (15%) reported simultaneous total loss of both. The median age of patients with any degree of smell disturbance was significantly lower than patients without (median 30.5 years (IQR 24 – 43.25) versus 41 years (IQR 28.25 – 52.75), *p* = 0.025). The median age of patients with any degree of taste disturbance was lower than those without, but did not reach statistical significance (median 34 years (IQR 24 - 45) versus 40 years (IQR 27.5 – 52.5) *p* = 0.174).

**Conclusion:**

Smell or taste dysfunction were present in our defined subgroup of patients. Further research is required in different population cohorts to build the evidence base for smell and taste dysfunction as clinical indicators of CoVID19 disease and to assess if these symptoms persist after disease resolution.

## Introduction

Clinical features of respiratory infections are potentially useful as diagnostic indicators of disease. [1] Infections have been identified as an important cause of olfactory and gustatory disorders and may result from damage of the olfactory epithelium. [2] Coronaviruses have previously been identified as causative pathogens in post-viral olfactory dysfunction. [3] SARS-CoV-2, a novel beta coronavirus, is a respiratory pathogen which causes CoVID19 disease. As of 24/07/2020, over 15 million SARS-CoV-2 infections have been recorded globally, resulting in over 600 000 deaths. [4].

O′ Donovan and colleagues have recently conducted a rapid review of the literature on the olfactory symptoms hyposmia (reduced sense of smell) and anosmia (complete loss of sense of smell) as clinical features of CoVID19. [5] They have recommended that clinicians assess olfactory sensation in patients with suspected CoVID19 to further establish this link.

At the time of our study, The European Centre for Disease Prevention and Control did not specifically delineate taste and smell disturbances as features of CoVID19. A recent update to that guidance now includes these symptoms in the case definition [6]. As such, our study investigated the prevalence of these features in a cohort of newly diagnosed COVID19 patients triaged to management in an outpatient setting through a telephone clinic. This was a rapid assessment undertaken in the context of the continually evolving nature of the CoVID19 pandemic.

## Methods

Our current institutional CoVID19 management pathway allows for patients (including healthcare workers) with mild disease and who do not require hospital admission to be isolated at home and managed through regular telephone consultation. Where disease progression is identified, patients are admitted for acute care management. On one single day (24/03/2020) we assessed a convenience sample of 46 patients (from a clinic list of 50 patients) to report their olfactory and gustatory sense function. This was measured on an adapted 5-point intensity scale, as previously used by Amézaga et al. [7], ranging from 1 (“no change”) to 5 (“very intense change”). Four of the 50 patients were uncontactable by telephone on the day. Our institutional review board (ref: 6164) and hospital research ethics committee (ref: 2020-04 CA 03) approved this study. Microsoft Excel and SPSS v.25 were used to collate and analyse the data. Counts and proportions were reported for categorical variables while medians and interquartile ranges were reported for continuous data. The Mann-Whitney U test was used to compare smell and taste disturbance scores between independent categories.

## Results

### Patient characteristics

The majority of our study cohort were female, were never smokers with a median age of 36.5 years (Table 1).

**Table I:** Patient characteristics

	*n*= 46
**Sex**	
Male, *n* (%)	19 (41.3)
**Age in years, median (IQR)**	36.5 (27–48)
**Days since symptom onset, median (IQR)**	6.5 (5–9.25)
**Smoking Status**^a^**, *n* (%)**	
Current	2 (4.3)
Ex-smoker	12 (26.1)
Never	31 (67.4)
**Co-morbidities**^b^**, *n* (% where stated)**	
Yes^c^	17 (37)
Asthma	7
Cancer	5
Cardiovascular disease	3
Hypothyroidism	1
Anaemia	1
Spina-bifida	1
Cerebrovascular disease	1
Sleep apnoea	1
No	27 (58.7)

IQR: interquartile range.

a Data missing for one patient.

b Data missing for two patients.

c Some patients had more than one co-morbidity.

### Symptoms of CoVID19, smell and taste disturbance scores

**Figure 1:**
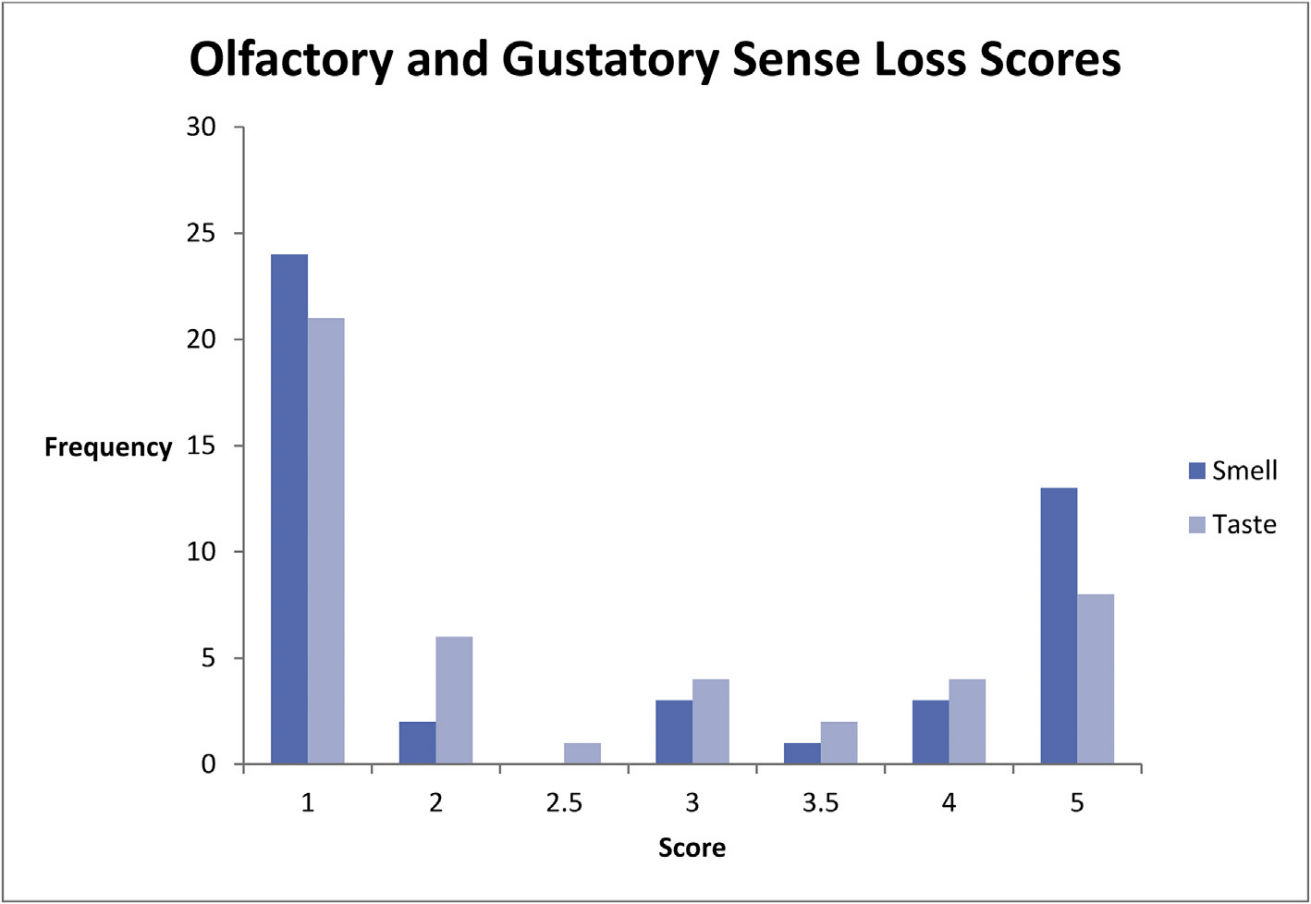
Patient reported scores of taste and smell disturbances.

Approximately half of patients reported some degree of olfactory (22/46, 48%) or gustatory (25/46, 54%) sense loss. Thirteen patients (28%) reported a complete loss of sense of smell, 8/46 (17%) reported a complete loss of sense of taste while 7/46 (15%) reported total loss of both (Figure 1).

Smell and taste scores were not significantly associated with gender, smoking status or presence of co-morbidities as assessed by the Mann-Whitney U test.

However, the median age of patients with any degree of olfactory disturbance was significantly lower than patients without [median 30.5 years (IQR 24–43.25) versus 41 years (IQR 28.25–52.75), *p* = 0.025] The median age of patients with any degree of gustatory disturbance was lower than those without, but did not reach statistical significance [median 34 years (IQR 24–45) versus 40 years (IQR 27.5–52.5) *p* = 0.174].

## Discussion

Our study has found that, in a cohort of patients with confirmed mild CoVID19 disease suitable for remote medical management, approximately half of them experienced new onset smell or taste dysfunction. Younger patients were disproportionally affected by smell, but not taste loss. To our knowledge, this is the first reported taste and smell disturbance data in CoVID19 patients from the Irish healthcare system.

The American Academy of Otolaryngology-Head and Neck Surgery has reported initial findings from its online Anosmia Reporting Tool. [8] Out of 237 patients, most reports originated in the United States. Anosmia was the initial symptom in 27% of cases and contributed to the patient seeking out CoVID19 testing in 40% of cases.

In their recent cross -sectional study of hospitalised patients with CoVID19 in Italy, Giacomelli *et al.* reported a loss of both olfactory and gustatory function in their cohort. [9] As with our study, they also found that olfactory and taste disturbances were more frequent in younger patients. Menni *et al.* reported that smell and taste disturbances were predictive for the presence of CoVID19 in their community-based study in the United Kingdom. [10] This study, however, appeared to combine smell and taste sense loss together as a single variable.

Our study is a retrospective analysis of a small cohort of patients with mild CoVID19 disease who were suitable for medical management through a regular telephone clinic at a large teaching hospital in Ireland. Our results may not be generalisable to other contexts and may not represent the presence of these features in patients with more severe CoVID19 disease. As such, the prevalence of smell and taste disturbances should be further explored in larger patient cohorts and with varying spectra of CoVID19 severity. A prospective design would enable analysis of the duration of smell and taste disturbances and whether these symptoms persist after resolution of disease. Further work should also investigate the prevalence of olfactory and gustatory disturbance in all patients being tested for CoVID19, in order to evaluate the diagnostic value of these features in predicting the presence of the disease.

## Credit author statement

Colm Kerr: conceptualization, methodology, investigation, data curation, formal analysis, writing (review and editing).

Gerry Hughes: conceptualization, methodology, data curation, formal analysis, writing (original draft preparation).

Louise McKenna: conceptualization, methodology, investigation, data curation, writing (review and editing).

Colm Bergin: conceptualization, resources, supervision, writing (review and editing), project administration.

## Transparency declaration

The lead author of this study declares that the manuscript is an honest, accurate, and transparent account of the study being reported; that no important aspects of the study have been omitted.

## Funding

This study was conducted in the normal course of the authors' work with no additional funding support.

## Competing interests

All authors declare no competing interests relevant to the publication of this research.
